# Pitstop‐2 and its novel derivative RVD‐127 disrupt global cell dynamics and nuclear pores integrity by direct interaction with small GTPases

**DOI:** 10.1002/btm2.10425

**Published:** 2022-10-19

**Authors:** Ivan Liashkovich, Sílvio Terra Stefanello, Reshma Vidyadharan, Günter Haufe, Alexander Erofeev, Peter V. Gorelkin, Vasilii Kolmogorov, Caren Rigon Mizdal, Alexander Dulebo, Etmar Bulk, Ian U. Kouzel, Victor Shahin

**Affiliations:** ^1^ Institute of Physiology II, University of Münster Münster Germany; ^2^ Organic Chemistry Institute, University of Münster Münster Germany; ^3^ National University of Science and Technology «MISiS» Moscow Russia; ^4^ Department of Chemistry Lomonosov Moscow State University Moscow Russia; ^5^ Bruker Nano GmbH, JPK BioAFM Business Berlin Germany; ^6^ University of Konstanz Constance Germany

**Keywords:** atomic force microscopy, cellular physiology, clathrin, nanomedicine, nuclear pores, pharmacology, small GTPases

## Abstract

Clathrin‐mediated endocytosis (CME) is an essential cell physiological process of broad biomedical relevance. Since the recent introduction of Pitstop‐2 as a potent CME inhibitor, we and others have reported on substantial clathrin‐independent inhibitory effects. Herein, we developed and experimentally validated a novel fluorescent derivative of Pitstop‐2, termed RVD‐127, to clarify Pitstop‐2 diverse effects. Using RVD‐127, we were able to trace additional protein targets of Pitstop‐2. Besides inhibiting CME, Pitstop‐2 and RVD‐127 proved to directly and reversibly bind to at least two members of the small GTPase superfamily Ran and Rac1 with particularly high efficacy. Binding locks the GTPases in a guanosine diphosphate (GDP)‐like conformation disabling their interaction with their downstream effectors. Consequently, overall cell motility, mechanics and nucleocytoplasmic transport integrity are rapidly disrupted at inhibitor concentrations well below those required to significantly reduce CME. We conclude that Pitstop‐2 is a highly potent, reversible inhibitor of small GTPases. The inhibition of these molecular switches of diverse crucial signaling pathways, including nucleocytoplasmic transport and overall cell dynamics and motility, clarifies the diversity of Pitstop‐2 activities. Moreover, considering the fundamental importance and broad implications of small GTPases in physiology, pathophysiology and drug development, Pitstop‐2 and RVD‐127 open up novel avenues.

## 1 INTRODUCTION

Clathrin‐mediated endocytosis (CME) is an essential gateway of material exchange and communication of eukaryotic cells with their environment.^1^ Its intimate involvement with a multitude of physiologic^2^, ^3^ and pathophysiologic^4^, ^5^, ^6^ processes has instigated an intensive search for small molecule compounds capable of inhibiting the clathrin‐mediated uptake. Among the most recent and potent inhibitors is Pitstop‐2, a compound which has been demonstrated to bind directly to the terminal β‐propeller domain of the clathrin heavy chain causing an arrest of coated pit dynamics.^7^ However, substantial effects other than inhibition of clathrin functions and CME, were subsequently reported by several groups including ours. In effect, the recommendation was expressly stated that Pitstop‐2 be used with caution, and that its effects should not be interpreted to conclude anything pertaining to the function of the N‐terminal domain of clathrin.^8^ First, Pitstop‐2 was shown to have an inhibitory effect on the clathrin‐independent endocytosis.^9^ Then, its ability to disrupt the clathrin function at kinetochores of dividing cells was reported.^10^ Subsequently, nonspecificity of the compound was demonstrated in a system where all four potential binding sites at the clathrin terminal domain were inactivated by site‐directed mutagenesis.^8^ This was followed by further experimental evidence we provided, which demonstrated drastic disruptive effects of Pitstop‐2 on the ultrastructure and functional integrity of nuclear pore complexes (NPCs), the mediators of all nucleocytoplasmic transport.^11^ NPCs are made up of multiple copies of 30 different types of proteins termed nucleoporins (Nups), a third of which is rich in phenylalanine‐glycine (FG) repeats, termed FG‐Nups, which control NPC transport and selectivity.^12^, ^13^, ^14^ Pitstop‐2 disrupted the structural configuration of NPCs and impaired the binding of the import receptor importin‐ß to FG‐Nups,^11^ an essential process for selective receptor‐mediated transport across NPCs.^13^

The effects of the inhibitor on the NPC functions were subsequently confirmed in an independent study.^15^ Some of the activities mentioned above were ascribed to the inhibition of nonendocytic functions of the clathrin heavy chain.^10^ Others, however, could not be attributed to the loss of known clathrin functions,^8^, ^11^ and alternative protein targets of Pitstop‐2 remained unexplored. The aim of this work is to identify the possible alternative targets for Pitstop‐2 which could clarify the reported activities.

## 2 RESULTS AND DISCUSSION

### 2.1 Synthesis and activity validation of a fluorescent derivative of Pitstop‐2, RVD‐127

To facilitate the identification of alternative, clathrin‐independent, protein targets of Pitstop‐2, we designed and synthesized a novel fluorescent derivative of the original inhibitor (lambdaEM ~520 nm),^7^ termed RVD‐127. It was synthesized on the basis of the procedure published for the original Pitstop‐2.^16^ Among a small library, a fluorescent derivative named RVD‐127 containing an *N*,*N*‐dimethylamino substituent in the 5‐position of the naphthyl ring and a MeO‐group instead of a para‐Br substituent in the styrene moiety was synthesized (Figure S1) and validated in diverse experiments. In addition, our docking studies with the crystal structure of the N‐terminal of clathrin reveal that RVD‐127 occupies the same binding site as Pitstop‐2 (Figures S2a,b). From these observations, we hypothesized that RVD‐127 will show a similar bioactivity as the lead structure.

**FIGURE 1:**
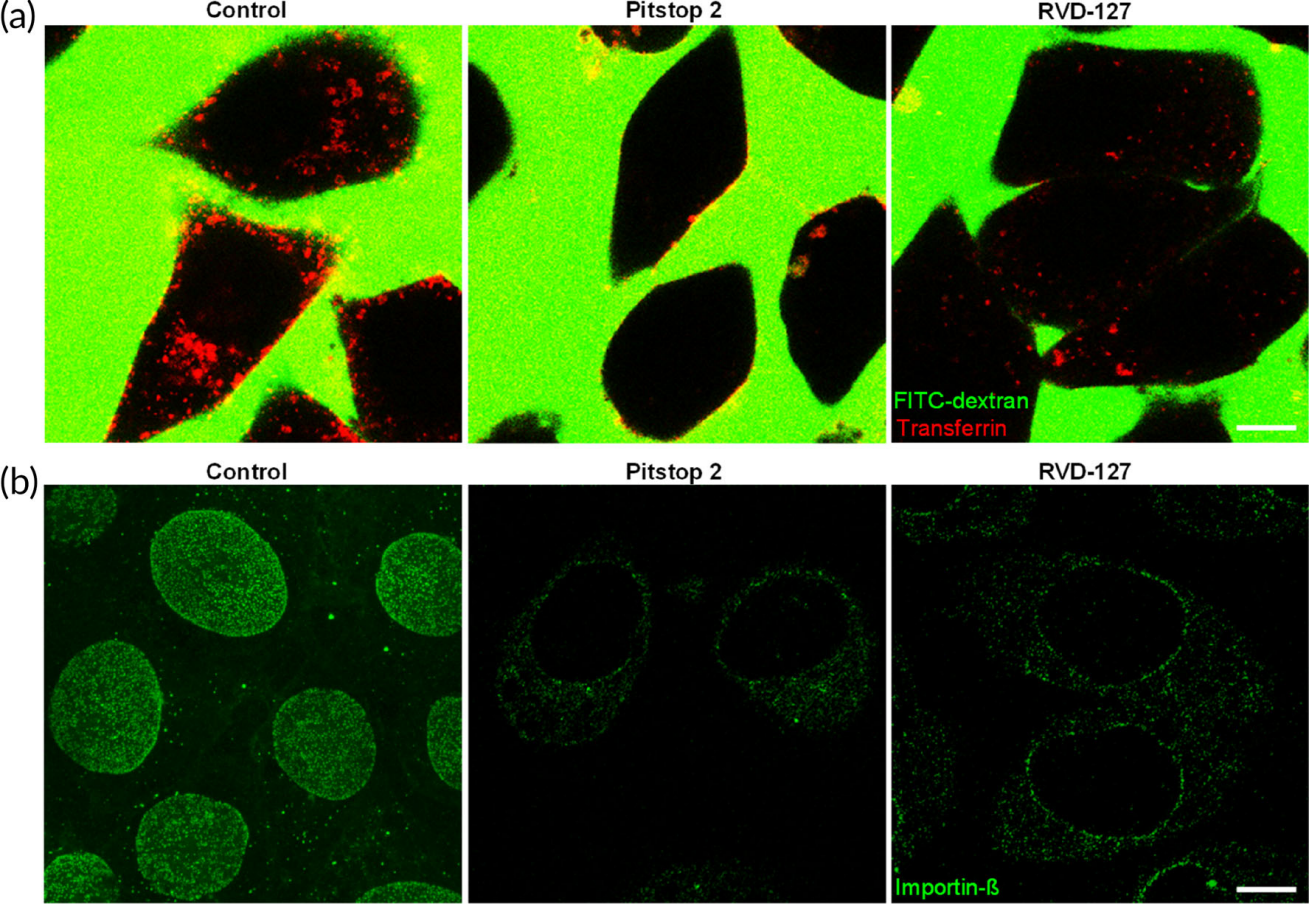
Experimental validation of the biological activities of the fluorescent derivative of Pitstop‐2, RVD‐127 with confocal fluorescence microscopy. (a) 100 μM RVD‐127 retains the ability to inhibit uptake of the classical substrate for clathrin‐mediated endocytosis, transferrin (red), to a similar extent as 30 μM Pitstop‐2. The medium of cells contains 70 kDa FITC‐dextran (green), which remains excluded from cellular uptake thereby delivering a negative image of the cells. Control cells are treated with the solvent of Pitstop‐2 and RVD‐127 (dimethyl sulfoxide; *N* = 5, and 60 or more cells each). (b) 30 μM Pitstop‐2 and 100 μM RVD‐127 are able to prevent association of importin‐β (Alexa‐488 labeled, green) with the nuclear pore complexes, and its intranuclear accumulation (*N* = 5, ≥60 cells each). Scale bars = 10 μm.

We first verified that the fluorescent RVD‐127 is able to reproduce the cell biological effects of Pitstop‐2. We then reasoned that it should enable us to visualize the spatial distribution of the targets within the cells. Figure 1 shows that RVD‐127 was able to significantly decrease the uptake of the classical substrate for CME transferrin in endothelial cells (Figure 1a). It also inhibited both the intrinsic association of importin‐β (Alexa‐488 labeled) with the FG‐Nups in NPCs, and thus its natural concentration in the nuclear envelope, as well as its nucleoplasmic translocation and accumulation (Figure 1b), albeit at higher effective concentration than the unmodified Pitstop‐2 (100 μM RVD‐127 vs. 30 μM Pitstop‐2).

In our previous study,^11^ to investigate whether Pitstop‐2 had an impact on the NPC interaction with the major active transport receptor importin‐β, we incubated digitonin‐permeabilized mammalian cells with bacterially expressed and Alexa‐488‐labeled importin‐β. To illustrate the specificity of the importin‐β binding, we performed a concomitant immunostaining with mAb414, a monoclonal antibody which specifically binds several FG‐Nups. As a result, we were able to quantify the extent of importin‐β binding to the NPCs in presence of Pitstop‐2 using the antibody signal as a normalization control. Confocal laser scanning microscopy revealed that importin‐β was not only able to bind to the NPCs of the digitonin‐permeabilized EA.hy926 but also accumulated in the nucleoplasm in presence of the Pitstop‐2 negative control or solvent.^11^ In presence of Pitstop‐2, the situation was markedly different. The binding of importin‐β to the NPCs was dramatically reduced while the mAb414 signal remained largely unaffected. Moreover, the intranuclear accumulation of importin‐β was largely abolished. Hence, the observations made with RVD‐127 in this study are consistent with our previous observations with the original inhibitor Pitstop‐2.^11^

These observations confirm that although the aforementioned chemical modifications of the original inhibitor apparently lower the affinity of RVD‐127 to its targets, they do not abrogate neither the intended inhibitory effect on CME nor the effect on NPC.

### 2.2 Pitstop‐2 and RVD‐127 cause dissociation of importin‐β from NPC by direct binding to the small GTPase ran

**FIGURE 2:**
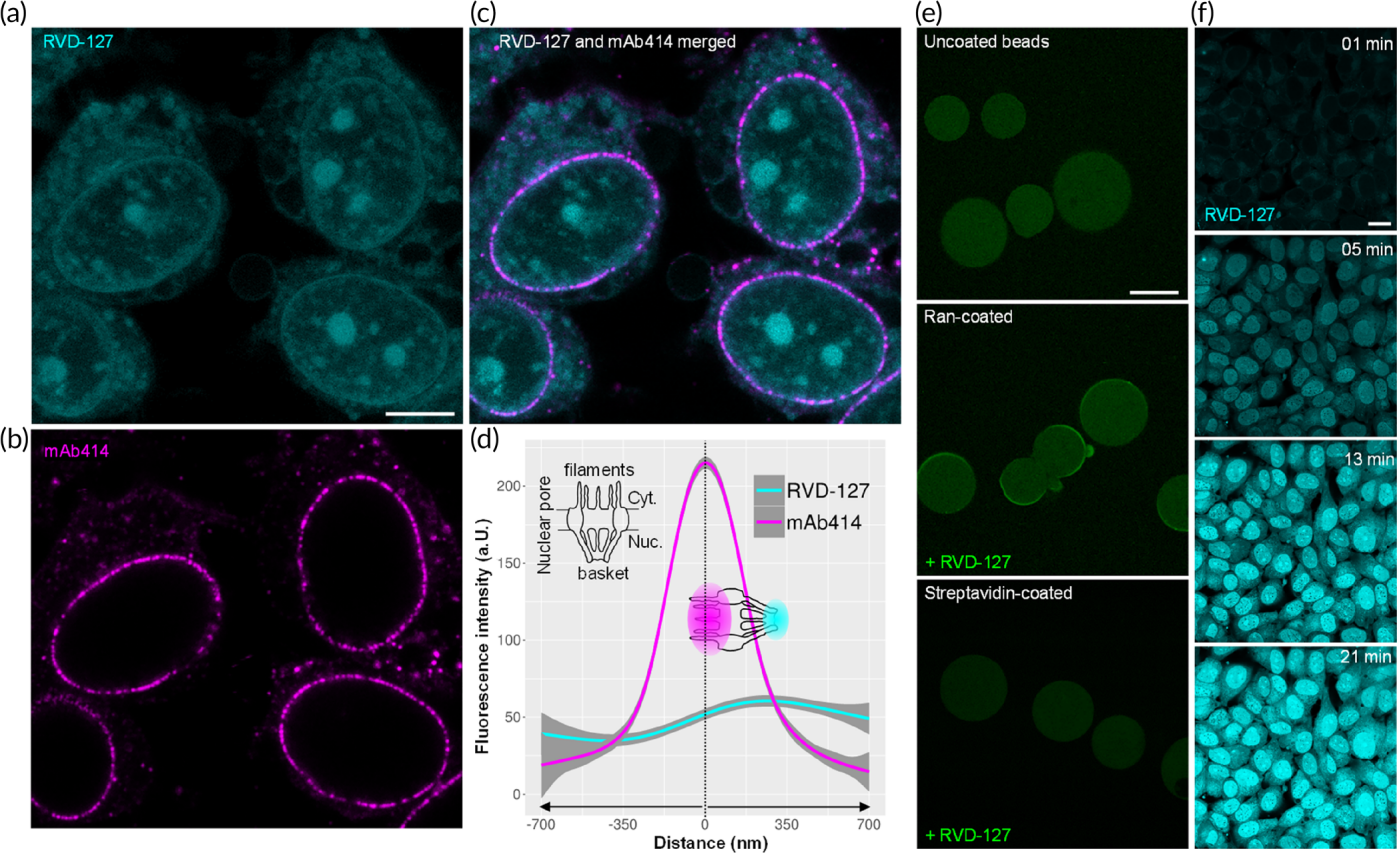
RVD‐127 disrupts selective nucleocytoplasmic transport across nuclear pore complexes (NPCs) by direct binding to the small GTPase Ran. (a) Subcellular distribution of the RVD‐127 (cyan) targets (*N* = 3, ≥60 cells each). Scale bar = 10 μm. (b) NPCs staining with mAb414 antibody (magenta), which stains phenylalanine‐glycine‐nucleoporins (FG‐Nups). (c) Merged image of (a) and (b). (d) Fluorescent staining with 100 μM RVD‐127 reveals a gradient of the distribution of its targets along the entire cytoplasmic (Cyt.) and nucleoplasmic (Nuc.) length of the NPC peaking at the nuclear basket 200 nm away from the FG‐nucleoporins labeled with mAb414. Three separate experiments were performed and the fluorescence intensity section profiles of 100 NPCs from 20 different cells were averaged and plotted (a.U., arbitrary units). (e) The gradient of the RVD‐127 staining within the NPC is clarified by its direct interaction with Ran GTPase. Heterologously expressed and purified recombinant Ran (Hexahistidin‐Tag) immobilized on Ni‐NTA agarose beads displays a characteristic staining of the bead edge when exposed to RVD‐127. Beads functionalized with other proteins (streptavidin, negative control) lack rim staining, which is indicative of a very limited interaction (*N* = 3). Scale bar = 100 μm. (f) Dynamics of intranuclear accumulation of RVD‐127 indicative of GDP‐like conformation of Ran induced by RVD‐127 (*N* = 3). Scale bar = 10 μm.

Having confirmed that RVD‐127 is able to reproduce the effects of the unmodified inhibitor, we performed confocal microscopy fluorescence imaging of cells stained with RVD‐127 to trace its binding sites. The aforementioned disruption of importin‐ß binding (Figure 1b) by the inhibitors implies that they may act on NPCs by directly or indirectly targeting FG‐Nups, which are known to mediate importin‐ß binding and selective NPC transport. FG‐Nups are particularly abundant inside the NPC channel, but they also occur elsewhere across the NPC. Fluorescence imaging analysis in Figure 2 shows RVD‐127 staining occurs throughout the cell, indicating that it targets an abundant and highly mobile component in the cell, which will be discussed later on. The analysis also reveals that despite the lack of immediate colocalization with the FG‐Nups specific antibody mAb414, RVD‐127 localizes along the entire NPC axis (Figure 2d). It was shown that multivalent binding of importins and exportins for different FG‐Nups might be comparable for most of FG‐Nups along the NPC axis (or at least it was not proven vice versa), and this affinity can change only upon cargo‐complex formation or its dissociation upon RanGTP binding. The RVD‐127 distribution pattern across the cytoplasmic and nucleoplasmic sides of the NPC correlates fairly well with the NPC dimensions and the distribution of FG‐Nups.^17^, ^18^ This implies that RVD‐127 targets a dynamic key player in bidirectional nucleocytoplasmic transport, closely associated with NPCs and importin‐ß, and the small GTPase Ran is an immediate candidate. The presence of the small GTPase Ran is essential throughout the multistep bidirectional receptor‐mediated transport cycle across the NPC as thoroughly demonstrated and explained in previous works.^19^, ^20^ Ran is also highly mobile and occurs throughout the cell.^21^ It shuttles between the cytoplasm and the nucleus in its guanosine diphosphate (GDP) and GTP‐bound states. A RanGTP:RanGDP gradient from the nucleus: cytoplasm drives importin‐β‐cargo directionality and powers the receptor‐mediated transport cycle.^19^, ^20^, ^21^ The gradient is generated by the spatial separation of the guanine nucleotide exchange factor and Ran GTPase activating protein, which reside in the nucleus and cytoplasm, respectively.^21^ In brief, a protein bearing nuclear localization signal is recognized in the cytosol by an import receptor such as importin‐α. Subsequent binding to importin‐β specifically targets the formed cargo‐receptor‐complex to the NPC and mediates the translocation through the NPC channel by interaction with FG‐Nups.^20^, ^22^ Binding of RanGTP to importin‐β inside the nucleus dissociates importin‐β and leads it back to the cytosol through the NPC channel.^19^, ^20^ RanGTP is hydrolyzed in the cytoplasm to RanGDP by the Ran GTPase activating protein, in particular at the cytoplasmic NPC filaments, releasing importin‐β for the next transport cycle.^13^, ^23^ Eventually, RanGDP returns to the nucleus where it is converted to RanGTP by the chromatin‐associated guanine nucleotide exchange factor RCC1.^21^ Ran is also required for importin‐β interaction with certain FG‐Nups,^19^ and the translocation of the importin‐cargo‐complex through the NPC.^24^ The cell physiological roles of Ran are not limited to the regulation of nucleocytoplasmic transport but extend to the regulation of diverse other pivotal functions,^25^ which explains the abundance of this soluble and mobile small GTPase throughout the cell, with an estimated 10^7^ copies per cell.^21^

We therefore asked if Ran was implicated in RVD‐127‐induced effects and tested whether RVD‐127 is able to directly associate with purified recombinant Ran. When Ran was immobilized on Ni‐NTA affinity beads and immersed in RVD‐127 solution in a bead halo assay,^26^ a distinct staining of the bead edge was readily detected confirming our hypothesis of direct interaction of RVD‐127 with small GTPase Ran (Figure 2e). Although the bead halo assay does not allow distinguishing between the GTP‐bound or GDP‐bound conformation of Ran, extended time‐lapse imaging of live cells treated with RVD‐127 shows gradual accumulation of the inhibitor, particularly within the nucleus (Figure 2f), implying that it induces a GDP‐bound conformation. In this state, the small GTPase is imported into the nucleus for subsequent nucleotide exchange as mentioned above.^21^ Another argument in favor of the assumed RVD‐127‐induced locking of Ran in the GDP‐bound state comes from affinity studies. The affinity of Ran is 10‐fold higher for GDP than GTP, whereas both forms of Ran have equal affinity to RCC1.^27^ Ran GTP is faster binding and much slower dissociating from Ran.^27^ Also, GTP affinity to Ran is higher than that to GDP, when Ran is in complex with importin‐β.^28^ Further evidence for Ran being a direct target of RVD‐127 and Pitstop‐2 comes from computational docking analysis of the inhibitors to the crystal structure of RanGDP (Figure S4). Taken together, we conclude that Ran is directly implicated in the Pitstop‐2/RVD‐127‐mediated effects on importin‐β dissociation from NPCs and on NPCs themselves, as observed in this study and our previous work.^11^

### 2.3 Pitstop‐2 and RVD‐127 disrupt global cell motility and mechanics

So far, we have shown that RVD‐127 faithfully reproduced the cell physiological effects of the unmodified inhibitor Pitstop‐2, and displayed a robust interaction with Ran in the bead halo assay. Ran was, thus, demonstrated to be a direct protein target of Pitstop‐2. However, we could not rule out other targets and, therefore, moved on to pinpointing potential protein targets of Pitstop‐2, without chemical modifications. This was to ensure that any identified target should not result from the chemical modification of the original inhibitor, in order to obtain the fluorescent derivative. We adopted a complimentary approach to Pitstop‐2 target search to verify the results obtained with RVD‐127, using the drug affinity responsive target stability (DARTS) assay (Figure S3).^29^ The underlying principle of the assay is based on the notion that specific binding of a small molecule inhibitor to its target protein stabilizes the target and, thus, reduces its susceptibility to proteolysis. We reasoned that if the effects of Pitstop‐2 on the NPCs can be observed in digitonin‐permeabilized cells or isolated nuclear envelopes,^11^ its binding to both the target and the off‐target proteins is likely to be retained when the cells are lysed completely. Sodium dodecyl‐sulfate polyacrylamide gel electrophoresis (SDS‐PAGE) separation of the cell lysates treated with progressively increasing concentration of a mixture of proteases in presence of the inhibitor vs solvent has revealed a distinct candidate band in the lysates supplemented with Pitstop‐2 (Figure S3).

Mass‐spectrometric analysis of the protein band protected from proteolysis has revealed that it was predominantly composed of several isoforms of actin (PDB accession number P60709). The result was reproduced in three independent experiments. This finding together with the originally observed “freezing” of the clathrin‐coated pit dynamics,^7^ upon of exposure of cells to Pitstop‐2, has prompted us to explore a possible effect of Pitstop‐2 on actin dynamics. Cytoskeletal actin dynamics is essential for endocytosis,^4^, ^30^ and if disrupted, whatever the cause may be, it should consequently give rise to “freezing” of highly dynamic cellular processes including endocytosis. This probably accounts for the “unspecific,” yet highly potent inhibitory effect of Pitstop‐2 on clathrin‐independent endocytosis, reported previously in clathrin knockdown cells.^9^ Another possible lead to a consequential actin dynamics inhibition by Pitstop‐2, as an implication of Ran inhibition, comes from previous experimental evidence demonstrating a close cross‐talk between the actin cytoskeleton and Ran‐mediated nuclear transport.^31^ Indeed, we have recently shown that reversible interference with the functional NPC integrity was promptly paralleled by drastic inhibition of cell motility and migration of both normal cells and highly aggressive lung cells.^32^

**FIGURE 3:**
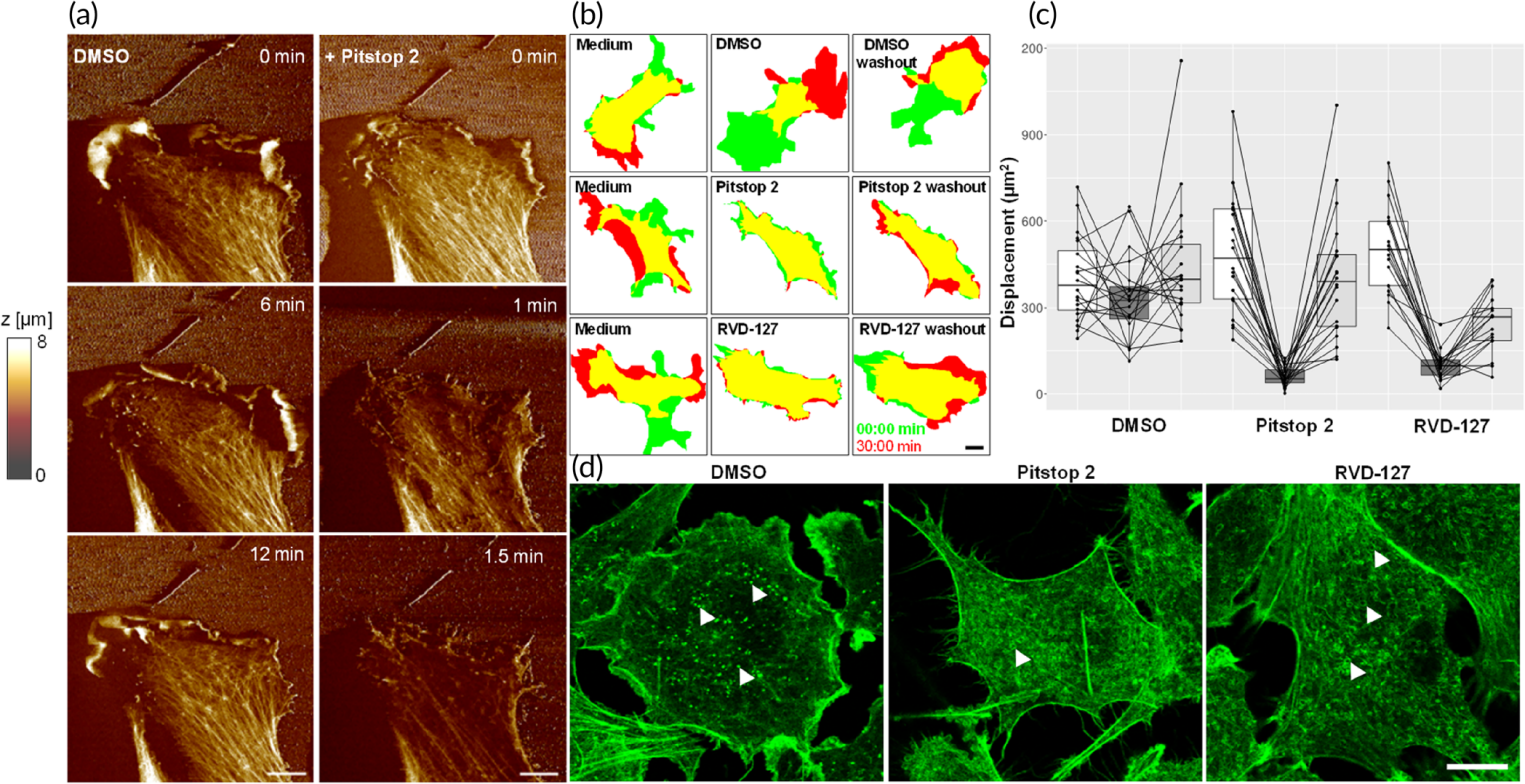
Pitstop‐2 and RVD‐127 disrupt actin dynamics and associated cell migration and mechanics. (a) High‐speed AFM imaging (frame rate = two images per minute) reveals that 7.5 μM Pitstop‐2 inhibits lamellipodial dynamics and causes gradual dismantling of cortical actin network within minutes of its application to living endothelial cells (bottom row, Video S1). (b,c) 50 μM RVD‐127 and 7.5 μM Pitstop‐2 induce an arrest of the endothelial cell motility. Quantification of the short‐term cell dynamics was performed by outlining the cell borders at time‐points 0 and 30 min for each condition (initial motility, inhibitor treatment, and inhibitor washout) and quantifying the cell area at time‐point 30 min which does not overlap with the initial cell position. The white, dark gray and light gray box shading in the plot corresponds to initial motility, inhibitor treatment, and inhibitor washout, respectively (*N* = 4, *n* = 20 cells for treatment with each type of inhibitor). Details about exact statistical analysis are given in the corresponding part in Experimental section. (d) Alexa‐488 phalloidin staining of EA.hy926 cells treated with 7.5 μM Pitstop‐2 or 50 μM RVD‐127 reveals ultrastructural alterations of actin cytoskeleton induced by the inhibitors. White arrowheads point to punctate foci of actin polymerization in dimethyl sulfoxide (DMSO)‐treated cells which are largely substituted by ring‐like structures in cells treated with Pitstop‐2 and RVD‐127 (*N* = 3, *n* ≥ 60 each). Scale bar = 10 μm.

Hence, based on the outcome of the DARTS assay we hypothesized that the arrest of clathrin‐coated pit dynamics might stem from a global impairment of actin‐based cell motility independently of clathrin terminal domain function. To test this hypothesis, we performed nanostructural and mechanical investigations on living cells before and after treatment with Pitstop‐2, as shown in Figure 3. High‐speed imaging with atomic force microscopy (AFM; frame rate = two images per minute) reveals that Pitstop‐2 dramatically reduces lamellipodial dynamics of endothelial cells within minutes of its addition (Figure 3a), and at significantly lower concentrations than those required for inhibition of transferrin uptake (7.5 vs. 30 μM for Pitstop‐2, and 50 vs. 100 μM for RVD‐127). The arrest of lamellipodial motility is closely followed by a gradual dismantling of the cortical actin network (Videos S1 and S2). The observations made with AFM investigations were corroborated with differential interference contrast (DIC) microscopy time lapse imaging, whereby cells were imaged for an initial period of 30 min, then exposed to Pitstop‐2, RVD‐127, or appropriate amount of the solvent (dimethyl sulfoxide [DMSO]) for the next 30 min, and finally, the compounds were washed out for the final 30 min of the time‐lapse imaging (Videos S3, S4, and S5). Quantification of the cell displacement during each stage of imaging (control, inhibitor treatment, and washout) reveals that both Pitstop‐2 and RVD‐127 are able to strongly inhibit overall cell motility (Figure 3b,c). The effect can then be reverted to an original level of motility during washout (Figure 3b,c). RVD‐127 induces a similar albeit not so rapidly reversible effect.

The drastic effect of both Pitstop‐2 and RVD‐127 on cell motility is paralleled by significant reorganization of actin cytoskeleton as revealed by phalloidin staining (Figure 3d). Two prominent effects on the ultrastructure of actin cytoskeleton are readily apparent. First, both Pitstop‐2 and RVD‐127 cause a depletion of F‐actin from lamellipodia consistent with the arrest of lamellipodial dynamics demonstrated by live‐cell imaging. The second effect is manifested as disappearance of the punctate actin foci probably associated with the sites of CME in cells treated with both inhibitors. Instead, ring‐like structures became increasingly prominent (Figure 3d).

**FIGURE 4:**
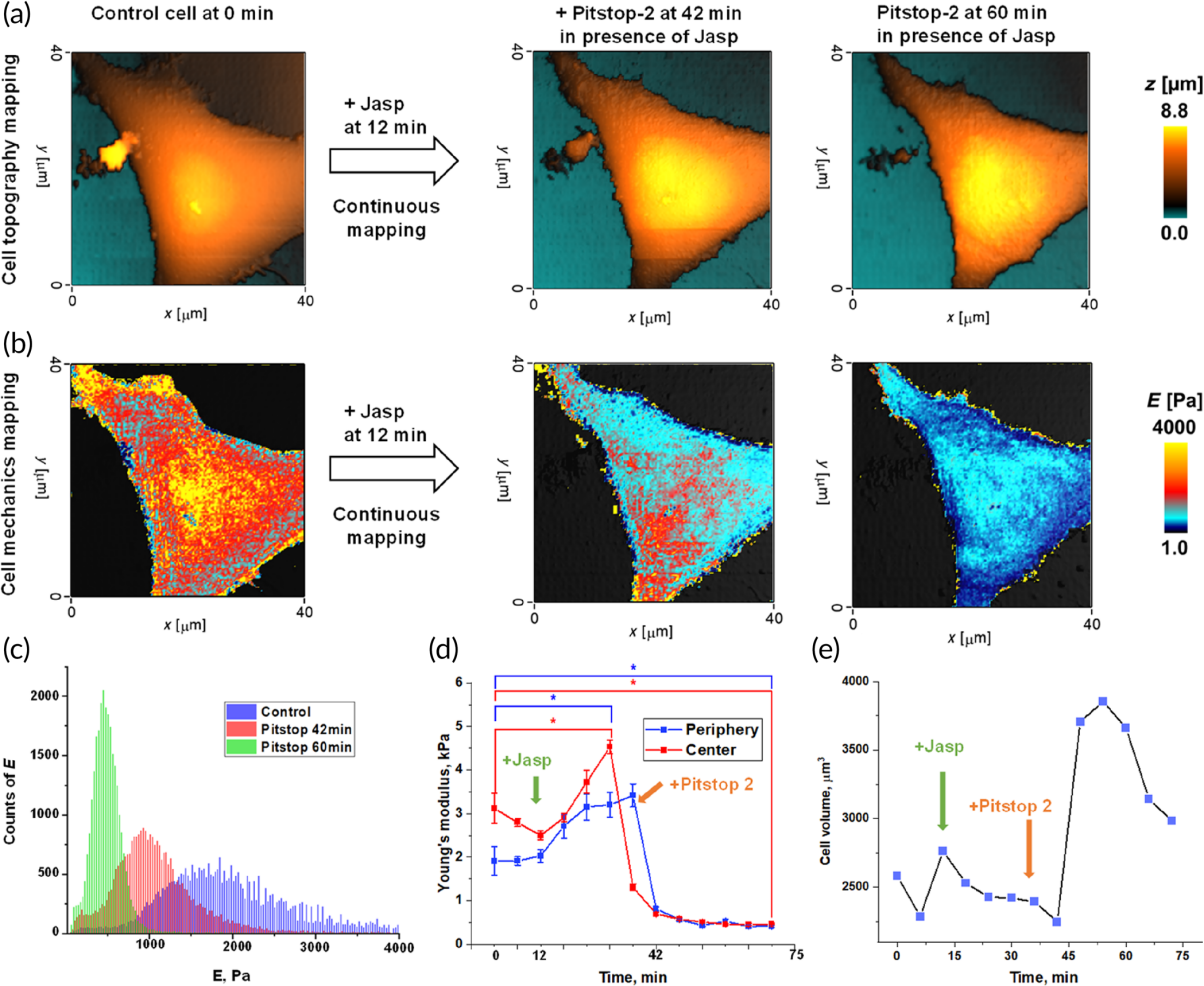
Pitstop‐2‐induced disruptive effects on the Actin cytoskeleton and global cell motility are paralleled by substantial increase in cell volume and decrease in cell stiffness: Simultaneous investigation of topography and mechanics of living EA.hy926 cells before and after treatment with Pitstop‐2, using Scanning Ion Conductance Microscopy (noncontact imaging and low‐stress mechanical properties measurements).^33^ (a) Continuous mapping of the topography of EA.hy926 cells before and after the addition of Pitstop‐2. At 12 min was added Jasplakinolide (Jasp), a commonly used Actin filament polymerizing and stabilizing compound. (b) Simultaneously to topography mapping, continuous mapping of the mechanics (stiffness: Elastic Young's modulus, E) of EA.hy926 cells before and after the addition of Pitstop‐2. (c) Young's modulus distribution of EA.hy926 before and after treatment with Pitstop‐2; (d) Dynamic of mean value of cell Young's modulus during treatment with Pitstop‐2. (e) Cell volume change during treatment with Pitstop‐2 (*N* = 3, 10 cells each, and in each cell, five different areas were analyzed for Young's modulus measurements). Asterisks indicate a statistically significant difference, *p* < 0.05, performed by Analysis of variance (ANOVA) test.

Consistently with the aforementioned Pitstop‐2‐induced disruptive effects on cytoskeletal actin and global cell dynamics, continuous mapping of the stiffness (elastic Young's modulus) of cells before and after Pitstop‐2, using Quantitative Nanomechanical Mapping (QNM) and Scanning Ion Conductance Microscopy (SICM),^33^ reveals that Pitstop‐2 destabilizes the global mechanics of cells, as demonstrated by the substantial decrease of the measured elastic Young's modulus summarized in Figure 4. The remarkable cell softening caused by Pitstop‐2 is paralleled by a remarkable increase of cell volume, which hints at depolymerization of actin, thereby giving rise to cell swelling.

### 2.4 Pitstop‐2 and RVD‐127 effect on actin dynamics is clathrin‐independent, and is mediated by direct interaction with the small GTPase Rac1

**FIGURE 5:**
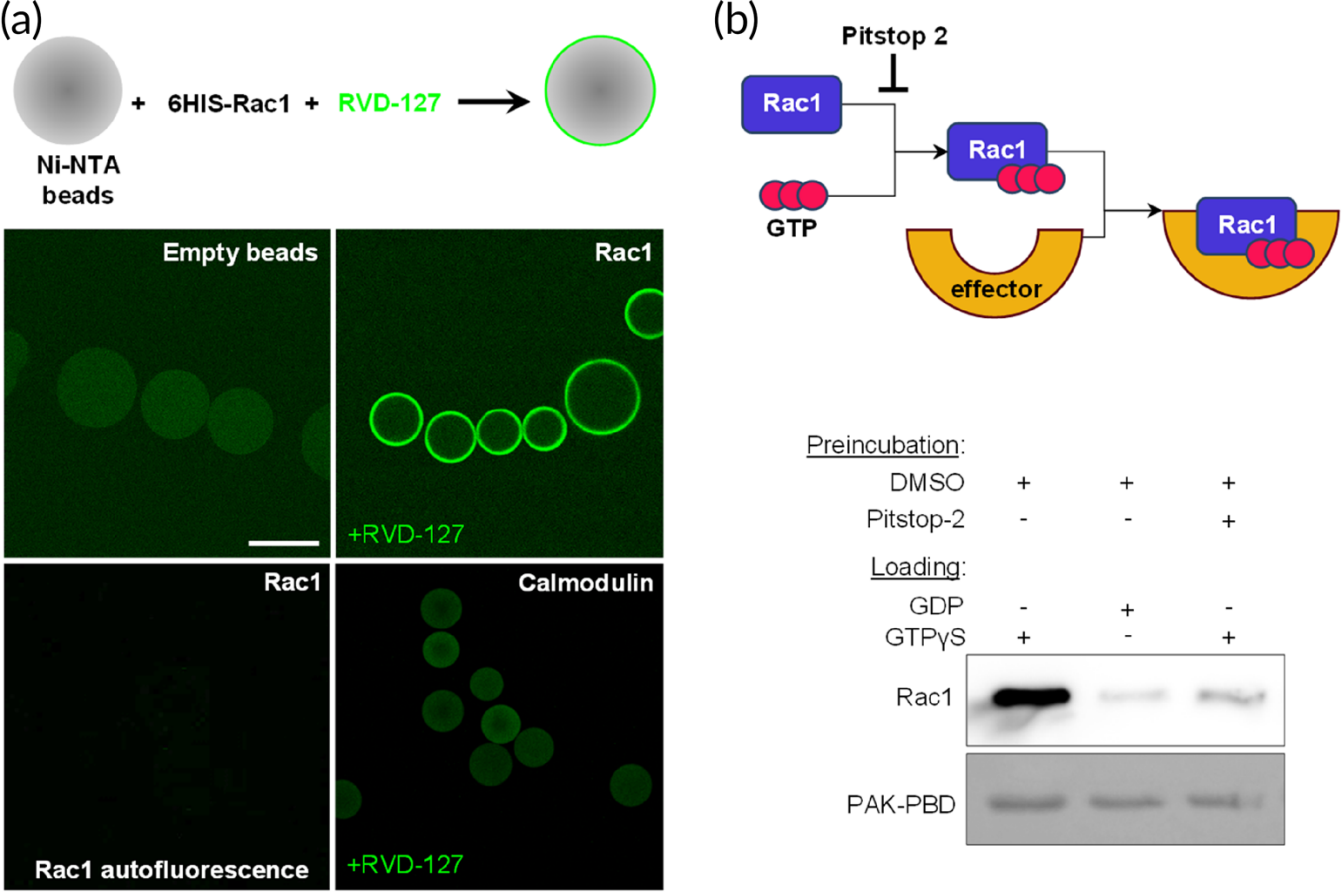
Pitstop‐2 and RVD‐127 directly interact with the small GTPase Rac1 and inhibit downstream signaling. (a) Direct interaction of RVD‐127 with heterologously expressed and purified recombinant small GTPases Rac1 (Hexahistidin‐Tag, 6His), immobilized on Ni‐NTA agarose beads, demonstrated by confocal imaging. Characteristic staining of the bead periphery is detectable for beads functionalized with small GTPases but not for the empty beads or unrelated proteins (calmodulin) used as controls. Scale bar = 100 μm. (b) Biochemical assay based on quantification of Rac1 interaction with its cognate effector PAK demonstrates that Pitstop‐2 abolishes this interaction even in presence of nonhydrolyzable GTP analog (GTPγS, right lane) which otherwise strongly promotes this interaction (left lane) compared with a much weaker binding in presence of GDP (middle lane; *N* = 3, 20 beads each). DMSO, dimethyl sulfoxide

To assess the possible involvement of CME inhibition in the observed drastic effects of Pitstop‐2 and RVD‐127 on cell dynamics, we monitored the uptake of transferrin at significantly lower concentrations than the required 30 μM. Reduced concentrations of Pitstop‐2 (7.5 μM) and RVD‐127 (50 μM), which lead to a nearly complete arrest of the overall cell motility paralleled by a readily apparent rearrangement of actin cytoskeleton, had only marginal effect on the CME of transferrin (Figure S4). Keeping in mind the direct binding of RVD‐127 to Ran, we hypothesized that the drastic and prompt effect of Pitstop‐2 and RVD‐127 on actin dynamics could be caused by interaction with another representative of the small GTPase superfamily Rac1. The latter is a key regulator of cellular dynamics,^34^ and was recently shown to increase cell stiffness,^35^ which probably explains the substantial decrease in cell stiffness we observed with QNM following exposure to Pitstop‐2. To test our hypothesis, a similar bead halo assay as performed with Ran, was also performed with Rac1, confirming a direct binding of RVD‐127 to Rac1, as shown in Figure 5. Control experiments where unrelated proteins were immobilized on agarose beads were tested under identical conditions show no characteristic stain of the bead edge. Auto fluorescence of Rac1 in absence of the compound under identical imaging conditions was not detected either (Figure 5a, bottom). If the inhibitor acts upstream of actin polymerization by blocking the activation of nucleators by Rac1 effectors,^36^ then its activity should be detected in a classical effector pull‐down assay. To corroborate this, we tested whether the chemically unmodified Pitstop‐2 is able to interfere with the primary function of Rac1 GTPase, its ability to interact with a downstream effector in its GTP‐bound state.^37^ This was indeed the case. When Rac1 was pre‐treated with Pitstop‐2 and then loaded with GTPγS, which should have induced effector‐binding as demonstrated in absence of Pitstop‐2, the binding of Rac1 to its downstream effector PAK was strongly inhibited (Figure 5b). In silico analysis of Pitstop‐2 binding to crystal structures of Ran^38^ and Rac1^39^ has further confirmed that Pitstop‐2 can indeed occupy the GDP‐binding pocket of both the Rac1 and Ran, and similar results were obtained with RVD‐127 (Figure S5). From this we conclude that Pitstop‐2, which was originally developed as an inhibitor of CME, directly interacts with a small GTPase Rac1 and induces a GDP‐bound conformation of this key regulator of cytoskeletal dynamics. The binding of Pitstop‐2 locks Rac1 in an inactive state and prevents its interaction with the downstream effectors. As a result, a reversible arrest of the lamellipodial dynamics and the overall cell motility is observed upon treatment with either the Pitstop‐2 or RVD‐127.

In summary, the newly developed fluorescent analog of Pitstop‐2, RVD‐127, has enabled identification of the novel targets of the inhibitor. We show that these include at least two members of small GTPase superfamily Ran and Rac1. Direct binding of RVD‐127 to Ran results in a comparable inhibition of importin‐β association with the NPCs as observed previously with chemically unaltered Pitstop‐2.^11^ We believe that the mechanistic explanation of inhibition of importin‐β binding to NPCs is akin to the previously described effect of the reversal of the RanGTP/GDP gradient.^40^ Pitstop‐2 or RVD‐127 induces a GDP‐like conformation of Ran, which is transported into the nucleus but is unable to undergo nucleotide exchange causing a collapse of the RanGTP/GDP gradient. As a result, intranuclear accumulation of Alexa‐488 labeled importin‐β is abolished. More surprising is the previously observed disruption of the overall structure of NPCs subjected to Pitstop‐2 treatment.^11^ This effect of Pitstop‐2 on the NPC ultrastructure points towards a more intimate involvement of Ran in organizing and maintaining the structural and functional state of the NPC, as experimentally demonstrated^41^ and reviewed in our previous works.^42^ Another intriguing fundamental implication of our findings is that the hypothesis of evolutionary relationship between membrane coats^43^ should probably be expanded. The mere structural resemblance of protein domains forming a relatively static structural framework of these coats needs to be integrated with the knowledge on more dynamic regulatory components exemplified by small GTPase molecular switches.

By extending the spatial and temporal observation framework of Pitstop‐2 activity from single clathrin‐coated pits to the whole cells observed over hours rather than seconds we found that the previously reported Pitstop‐2‐induced “freezing” of the clathrin‐coated pits^7^ is probably a drastic, albeit reversible arrest of the overall cell motility caused by Pitstop‐2 interaction with Rac1. The effect of the inhibitor on the cell motility is manifested at significantly lower concentration of Pitstop‐2 (7.5 μM) than the inhibition of CME (30 μM). This speaks strongly against the possibility that the defect of cell motility is caused by a disrupted clathrin‐mediated integrin recycling.^44^

## 3 CONCLUSIONS

Pitstop‐2 is a potent inhibitor of CME, but its inhibitory activities are not restricted to CME. This study sheds light onto the seemingly unspecific inhibitory effects of Pitstop‐2, reported on previously.^8^, ^9^, ^11^ The diverse inhibitory effects share a common theme, which is the requirement of small GTPases, be it directly or indirectly. We demonstrated here that Pitstop‐2 exhibits particularly high pharmacological efficacy as an inhibitor of small GTPases, requiring only approximately one‐fourth of the concentration necessary for CME inhibition. The ability of Pitstop‐2 to reversibly decouple the actin polymerization from the clathrin‐coated pits might be instrumental for generating new insights into the interplay between CME and actin cytoskeleton dynamics. The novel derivative RVD‐127, designed in this study, faithfully reproduces the effects of its original inhibitor, albeit at higher concentrations. The development of potent inhibitors of small GTPases is highly attractive, much sought after goal, for numerous clinical and biomedical applications owing to the paramount importance of small GTPases as molecular switches of diverse key physiological processes throughout the life cycle.^45^, ^46^ Therefore, the addition of Pitstop‐2 and RVD‐127 to the array of small molecules capable of modulating the function of small GTPases opens up new perspectives for further development. Although both inhibitors might be able to target additional small GTPases, their differential activity on Ran and Rac1 holds promise for chemical fine‐tuning aimed at further increasing the specificity towards a given member of the small GTPase superfamily. The fact that both Pitstop‐2 and RVD‐127 are reversible inhibitors offers a key advantage. The drastic inhibitory effects of Pitstop‐2 and RVD‐127 on global cell motility, on which most cellular activities heavily rely, render them particularly attractive candidates as potent anticancer and antiviral drugs among many other clinical applications. Another highly attractive potential future application of Pitstop‐2 and RVD‐127 could be the disruption of NPC integrity and nucleocytoplasmic transport. This also is highly desired and in the focus of recent drug design studies and future perspectives for diverse clinical applications including highly malignant cancer among many other severe and widespread pathologies.^47^, ^48^, ^49^, ^50^, ^51^

## 4 MATERIALS AND METHODS

### 4.1 Cell culture

EA.hy926 endothelial cells^52^ (kindly provided by Cora‐Jean Edgell, University of North Carolina, USA) were cultured at 37°C, 5% CO_2_, and 100% humidity in minimal essential medium (DMEM; Invitrogen Corp., Karlsruhe, Germany) containing 1% nonessential amino acids, 1% MEM vitamins (Invitrogen) and 10% fetal calf serum (PAA Clone, Coelbe, Germany).

### 4.2 Synthesis of RVD‐127

To a suspension of pseudothiohydantoin (1.5 g, 13.23 mmol) in DMF was added NaH (318 mg, 13.23 mmol) portionwise and the resultant milky white suspension was allowed to stir at room temperature for 30 min (Figure S1). Dansyl chloride (1.0 g, 4.41 mmol) was added portionwise to the stirring solution and the reaction mixture was allowed to stir at room temperature overnight. After the completion of the reaction, the mixture was quenched with slow addition of 1 M HCl. A pale yellow solid was isolated using vacuum filtration which was then washed with 1 M HCl, water, and ether and was dried under high vacuum to obtain pale yellow solid. Yield: 983 mg (64%).

Mp: 159°C–160°C; TLC (EtOAc:cyclohexane, 50:50 vol/vol): *R*_f_ = 0.39; ^1^H NMR (300 MHz, DMSO‐*d*_6_): δ 8.50 (d, *J* = 8.5 Hz, 1H), 8.24 (d, *J* = 8.4 Hz, 2H), 7.64 (ddd, *J* = 16.0, 8.5, 7.6 Hz, 2H), 7.26 (d, *J* = 7.2 Hz, 1H), 4.05 (s, 1H), 2.82 (s, 5H); ^13^C NMR (75 MHz, DMSO‐*d*_6_): δ 173.8, 173.0, 151.4, 135.9, 130.2, 129.3, 129.0, 128.1, 123.64, 119.5, 115.3, 45.1, 35.3; HRMS: *m/z* [M + H]^+^ calcd. For C_15_H_15_N_3_O_3_S_2_, 350.0628; found, 350.0622.

### 4.3 RVD‐127 = 5‐(dimethylamino)‐*N*‐[5‐(4‐methoxybenzylidene)‐4‐oxo‐4,5‐dihydrothiazol‐2‐yl]naphthalene‐1‐sulfonamide (5)

To a suspension of **3** (900 mg, 2.6 mmol) in absolute ethanol was added *p*‐anisaldehyde **(4)** (531 mg, 3.9 mmol) followed by 3 drops of benzoic acid/piperidine catalyst mix. This mixture was heated at 120°C using a 200 W microwave radiation for 20 min. The mixture was then allowed to cool to room temperature and subsequently cooled in an ice bath for 30 min. The resulting yellow solid was isolated by filtration, washed with ether and dried under high vacuum to obtain yellow solid. Yield 671 mg (55%).

Mp: 239°C–241°C (decomp.); TLC (EtOAc:cyclohexane, 60:40 vol/vol): *R*_f_ = 0.48; ^1^H NMR (400 MHz, DMSO‐*d*_6_): δ 8.50 (dt, *J* = 8.6, 1.1 Hz, 1H), 8.28 (ddd, *J* = 9.2, 7.8, 1.1 Hz, 2H), 7.75–7.57 (m, 5H), 7.31–7.21 (m, 1H), 7.18–7.09 (m, 2H), 3.84 (s, 3H), 2.82 (s, 6H); ^13^C NMR (101 MHz, DMSO): δ 161.3, 151.4, 135.9, 132.4, 130.3, 129.20, 128.9, 128.2, 127.9, 125.4, 123.6, 119.3, 115.2, 115.0, 114.5, 55.5, 45.1, 43.7, 22.2; HRMS: *m/z* [M + Na]^+^ calcd. For C_23_H_21_N_3_O_4_S_2_, 490.0866; found, 490.0866.

### 4.4 Drug affinity responsive target stability

The assay was performed essentially as described previously.^29^ Briefly, the cells were cultured to a confluent state, washed with PBS and lysed using M‐PER lysis buffer (Thermo Fischer Scientific GmbH, Schwerte, Germany). Protein concentration was determined using BCA‐assay kit (Thermo Fischer Scientific GmbH) and adjusted to 1 mg/ml. The lysate was split into two equal portions which were supplemented either with Pitstop‐2 (Abcam, plc., Cambridge, UK) (355 μg/mg protein) or with a corresponding amount of solvent (DMSO). The mixtures were equilibrated with the compounds for 1 h at room temperature and each was split into seven equal portions. Each portion was supplemented with a corresponding amount of Pronase (Roche, Mannheim, Germany) and proteolysis was carried out for 30 min at room temperature. Reaction was stopped by precipitating the proteins with acetone. Precipitated proteins were dissolved in 1× Laemmli SDS loading buffer and separated by linear 10% SDS‐PAGE with subsequent Coomassie staining. Candidate band was excised out of the gel and subjected to mass spectrometry identification.

### 4.5 DIC, AFM, and confocal imaging

For short‐term 90‐min cell migration experiments the cells were cultivated in μ‐Slides I (ibidi GmbH, Martinsried, Germany) for 48 h. One hour prior to imaging, full DMEM containing serum and antibiotics was replaced with serum‐free and antibiotics‐free medium and incubated at 37°C in 5% CO_2_. The time‐lapse imaging was performed using Observer D1 (Carl Zeiss AG, Oberkochen, Germany) with the frame rate of one image every 30 s. After 30 min, cells were subjected to treatment with 7.5 μM Pitstop‐2, 50 μM of RVD‐127, or the equivalent amount of DMSO‐containing medium. The inhibitors were washed out after 30 min with the serum‐containing and antibiotics‐containing DMEM and the cells were imaged for further 30 min.

For AFM and confocal imaging, the cells were cultured in 30 mm glass‐bottom Petri dishes (WillCo Wells B.V., Amsterdam, The Netherlands). Prior to drug treatment, the cells were pre‐incubated with the serum‐free and antibiotics‐free DMEM as described above. AFM imaging of the Pitstop‐2 effect on lamellipodial dynamics was carried out using Bioscope Resolve AFM (Bruker Inc., Santa Barbara, California) in fast tapping mode, using the PFQNM‐LC‐A‐CAL scanning probe (Bruker, Camarillo, California), at the frame rate of two images per minute. The transferrin (Thermo Fischer Scientific GmbH) uptake and importin‐β binding inhibition experiments were performed essentially as described previously.^11^ The inhibitory concentrations of Pitstop‐2 and RVD‐127 were 30 and 100 μM, respectively. NPC/RVD‐127 co‐staining was performed as reported previously^11^ with a minor modification. Digitonin‐permeabilized, mAb414‐stained cells were supplemented with 100 μM of RVD‐127 prior to confocal imaging.

### 4.6 Scanning ion‐conductance microscopy

Noncontact topographic imaging and low‐stress mechanical property measurements were performed using SICM manufactured by ICAPPIC (ICAPPIC Limited, UK). EA.hy926 cells were cultured on 35‐mm Petri dish. Cells were gently washed with Hank's solution (Gibco, USA) before scanning procedure. Nanopipettes with typical radius 35–40 nm were fabricated using a P‐2000 laser puller (Sutter Instruments, USA) from borosilicate glass capillaries (O.D. 1.2 mm, I.D. 0.90 mm, 7.5‐cm length) with the following programs Heat 310, Fil 3, Vel 30, Del 160, Pul 0, Heat 330, Fil 3, Vel 25, Del 160, and Pul 200. For all SICM measurements, a three‐set point mode was used. A noncontact topographic image was obtained at an ion current decrease of 0.5% and further two images were obtained at an ion current decrease of 1% and 2% corresponding to membrane deformations produced by intrinsic force at each setpoint (Kolmogorov et al., Nanoscale, 2021). After two measurements of topography and Young's modulus of single EA.hy926 cell Jasplakinolide were added to Petri dish in final concentration 50 nM. Then, the cell was continuously scanned within 24 min every 6 min. In the end, Pitstop‐2 was added to solution and cell was scanned within 24 min every 6 min.

### 4.7 Pitstop‐2 and RVD‐127 binding to small GTPases

Computational docking of Pitstop‐2 and RVD‐127 to the crystal structure of RanGDP (PDB code 3GJ0) was performed using AutoDockVina^53^ software to determine the putative binding sites of Pitstop‐2 and RVD‐127. Prior to docking the inhibitors GDP molecule was removed from the structure. For docking the small molecules to Rac1, the crystal structure of Rac1‐GDP complexed with Arfaptin (PDB Code 1I4D) was used due to the lack of a crystal structure of Rac1 bound to GDP. The Rac1‐GDP model was predicted with help of the modeling web portal Phyre2.^54^ Docking results were analyzed using AutoDockTools 1.5.6^55^ and the images were prepared using VMD 1.9.2.^56^ For the in vitro binding of RVD‐127 to recombinant GTPases, Rac1 (Cytoskeleton, Inc., Denver, USA) or Ran (generous gift of Ariberto Fassati) were immobilized on Ni‐NTA affinity beads (Qiagen, Hilden, Germany) and incubated in 100 μM of RVD‐127 in HEPES‐buffered solution (90 mM KCl, 10 mM NaCl, 2 mM MgCl_2_, 10 mM HEPES, pH 7.4). Confocal imaging of the beads was performed using Leica SP8 (Leica, Wetzlar, Germany). In vitro inhibition of the Rac1 binding to its downstream effector was performed using the Rac1 activation assay kit (Cytoskeleton) per manufacturer's instructions.

### 4.8 Data analysis

Data analysis of colocalization of RVD‐127 and mAb414 staining was performed essentially as described previously for co‐localization of Importin‐β and mAb414 (Covance, Münster, Germany).^11^ Three separate experiments were performed and the fluorescence intensity section profiles of 100 NPCs from 20 different cells were averaged and plotted with loess smoothing. Quantification of the short‐term cell dynamics was performed by outlining the cell borders at time‐points 0 and 30 min for each condition (initial motility, inhibitor treatment, inhibitor washout) and quantifying the cell area at time‐point 30 min which does not overlap with the initial cell position. Data were analyzed and visualized using R version 3.3.1^57^ and R package ggplot2.^58^ Linear Mixed Effects analysis was performed in nlme R package^59^ to investigate the relationship between experimental condition (control, treatment, washout) and the type of inhibitor (DMSO, Pitstop‐2 and RVD‐127). Square root transformation of the data was applied to stabilize the variance. Experimental condition and the type of inhibitor were set as fixed effects with interaction in the model, and individual cells were set as random effects. The model also accounted for different variance in each type of the inhibitor. Statistical significance of the fixed effects (type of the inhibitor and experimental condition) was finally assessed with ANOVA function. The effect of type of the type of the inhibitor (*F* = 12.77, *p* < 0.0001) and experimental condition (*F* = 104.47, *p* < 0.0001) as well as of their interaction (*F* = 17.88, *p* < 0.0001) were found to be highly significant. Model validation was performed by constructing QQ‐plot of the residuals, residuals versus fitted values and each explanatory variable (experimental condition, type of the inhibitor). Visual inspection did not reveal any obvious violations of the assumptions of normality and homogeneity. Other experiments were performed at least three times with representative images included in the figures.

### 4.9 Code availability

R script written to analyze the data using linear mixed effects model and its validation can be provided upon request.

### 4.10 Statistical analysis

Each experimental condition was repeated at least three times. Data are presented as mean values ± standard error of the mean. Results are considered as statistically significant at the probability level *p* < 0.05. The details regarding the number of experiments and analyzed cells, applied statistical tests and *p* values are specified in the corresponding parts.

## AUTHOR CONTRIBUTIONS

**Ivan Liashkovich:** Conceptualization (equal); data curation (equal); formal analysis (equal); funding acquisition (equal); investigation (equal); methodology (equal); validation (equal); visualization (equal). **Sílvio Terra Stefanello:** Formal analysis (supporting); software (supporting); validation (supporting). **Reshma Vidyadharan:** Formal analysis (equal); investigation (equal); methodology (equal); software (equal); validation (equal); visualization (equal). **Günter Haufe:** Conceptualization (equal); funding acquisition (equal); project administration (equal); resources (equal); supervision (equal). **Alexander Erofeev:** Investigation (supporting); methodology (supporting); visualization (supporting). **Peter V. Gorelkin:** Investigation (supporting); methodology (supporting); visualization (supporting). **Vasilii Kolmogorov:** Investigation (supporting); methodology (supporting); visualization (supporting). **Caren Rigon Mizdal:** Software (supporting); validation (supporting). **Alexander Dulebo:** Investigation (supporting); methodology (supporting); validation (supporting). **Etmar Bulk:** Investigation (supporting); validation (supporting). **Ian U. Kouzel:** Investigation (supporting); validation (supporting). **Victor Shahin:** Conceptualization (lead); funding acquisition (lead); methodology (lead); project administration (lead); resources (lead); supervision (lead); writing – original draft (lead); writing – review and editing (lead).

## CONFLICT OF INTEREST

The authors declare no competing financial interest.

### PEER REVIEW

The peer review history for this article is available at https://publons.com/publon/10.1002/btm2.10425.

## Supporting information


**Appendix S1** Supporting Information

**Video S1** Atomic force microscopy imaging of an endothelial cell dynamics prior to exposure to Pitstop‐2. Framerate = two images per minute

**Video S2** Atomic force microscopy imaging of an endothelial cell dynamics upon exposure to Pitstop‐2. Lamellipodial arrest and gradual dismantling of cortical actin network are readily apparent. Framerate = two images per minute

**Video S3** DIC imaging of an endothelial cell dynamics prior, during and after wash‐out of DMSO (dimethyl sulfoxide, solvent for Pitstop‐2 and RVD‐127). Framerate = two images per minute

**Video S4** DIC imaging of an endothelial cell dynamics prior, during and after wash‐out of Pitstop‐2. Framerate = two images per minute

**Video S5** DIC imaging of an endothelial cell dynamics prior, during and after wash‐out of RVD‐127. Framerate = two images per minute

## Data Availability

Data available on request from the authors.
