# Supplementary material for: Pitstop‐2 and its novel derivative RVD‐127 disrupt global cell dynamics and nuclear pores integrity by direct interaction with small GTPases
Source: Bioeng Transl Med. 2022 Oct 19;8(4):e10425. doi: 10.1002/btm2.10425 (PMC10354767; doi:10.1002/btm2.10425)
Supplement: Supplementary file 1 — Appendix S1 Supporting Information [file BTM2-8-e10425-s005.docx]

# Supporting Information

**Pitstop-2 and its novel derivative RVD-127 disrupt global cell dynamics and nuclear pores integrity by direct interaction with small GTPases**

*Ivan Liashkovich‡, Sílvio Terra Stefanello‡, Reshma Vidyadharan‡, Günter Haufe, Alexander Erofeev, Peter V. Gorelkin,Vasilii Kolmogorov, Caren Rigon Mizdal, Alexander Dulebo, Etmar Bulk, Ian U. Kouzel, Victor Shahin**

‡Equally contributed

Dr. I. Liashkovich, Dr. E. Bulk, Dr. S. T. Stefanello, Dr. C. R. Mizdal and Prof. Dr. V. Shahin Institute of Physiology II

University of Münster

Robert-Koch-Str. 27b, 48149 Münster, Germany.

E-mail: shahin@uni-muenster.de

Dr. R. Vidyadharan and Prof. Dr. G. Haufe Organic Chemistry Institute

University of Münster

Corrensstrasse 40, 48149 Münster, Germany

Dr. Ian U. Kouzel University of Konstanz

Universitaetsstraße 10, 78464 Konstanz

Dr. A. Erofeev

National University of Science and Technology «MISiS» 119049 Moscow, Russia

And

Department of Chemistry, Lomonosov Moscow State University, 119991 Moscow, Russia

Dr. P. V. Gorelkin and V. Kolmogorov

National University of Science and Technology «MISiS» 119049 Moscow, Russia

Dr. A. Dulebo

Bruker Nano GmbH, JPK BioAFM Business Am Studio 2D

12489 Berlin, Germany

**Figure S1:**


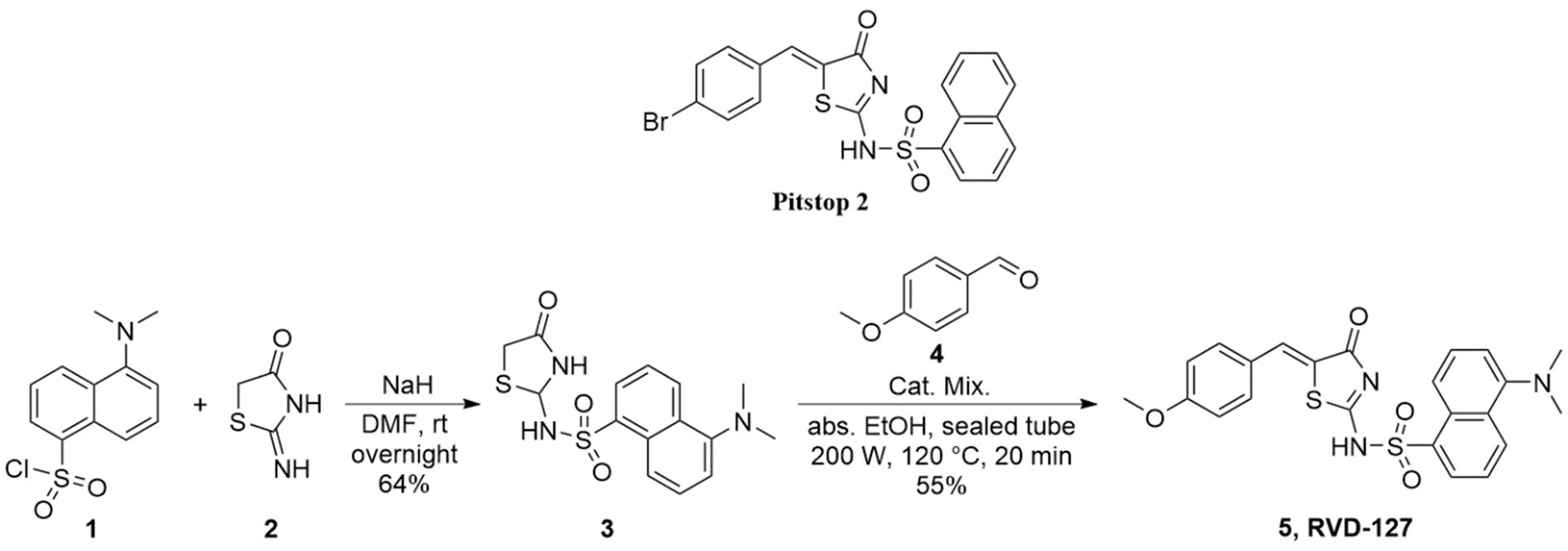


**Figure S1**. Synthesis of RVD-127, a fluorescent derivative of Pitstop 2. 1 = 1- Naphthalenesulfonylchloride (dansyl chloride). 2 = Pseudothiohydantoin. 3 = 5-(Dimethylamino)-N- (4-oxo-4,5-dihydrothiazol-2-yl)naphthalene-1-sulfonamide. 4 = p-Anisaldehyde. 5 = 5- (Dimethylamino)-N-[5-(4-methoxybenzylidene)-4-oxo-4,5-dihydrothiazol-2-yl]naphthalene-1- sulfonamide. Abs. EtOH = Absolute Ethanol.

**Figures S2a and S2b: Docking studies**

The crystal structure of the N-terminal domain of clathrin complexed with Pitstop-2, obtained from PDB (Protein Data Bank), was applied for the docking studies using the software AUTODOCK VINA (PDB ID 4G55) [1]. Pitstop 2 was removed using GAUSSVIEW 4.1.2 and a protein relaxation using CHARMM27 was performed [2]. This structure was then converted to PDBQT format (Q = partial charge, T = autodock type) for calculations using AUTODOCK VINA [3]. The Pitstop-2 was then re-docked into the protein to validate the docking software. It was observed that the lowest energy structure occupied the same binding site as the experimentally determined ligand.

Then the fluorescent RVD-127 was docked in the receptor, and it was observed that this ligand occupies the same binding site. An overlay of the original Pitstop-2 (yellow) with RVD-127 (red) in the binding site is shown in Figure S2a.


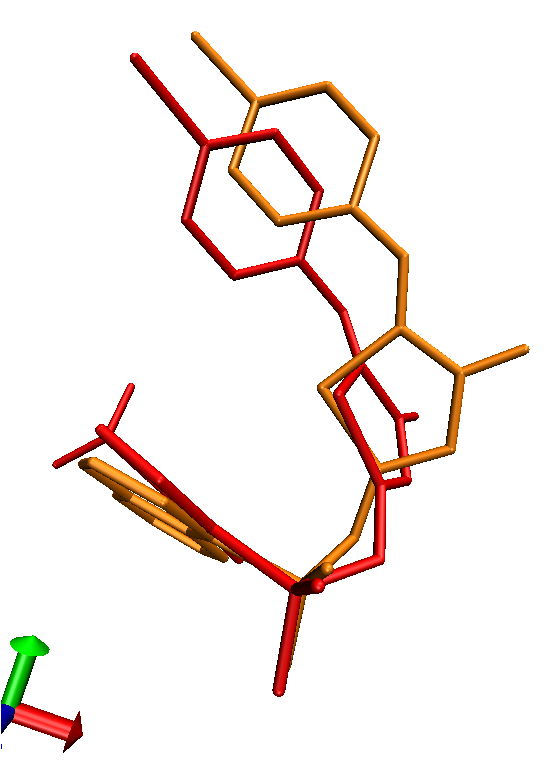


**Figure S2a.** An overlay of Pitstop-2 (yellow) and the fluorescent analogue RVD-127 (red).

[1] H. Bulut, L. von Kleist, W. Saenger, V. Haucke, *Cell* **2011**, *146*, 471-484.

[2] D. B. Kitchen, H. Decornez, J. R. Furr, J. Bajorath, *Nat. Rev. Drug Discov.* **2004**, *3*, 935–949.

[3] G. M. Morris, R. Huey, W. Lindstrom, M. F. Sanner, R. K. Belew, D. S. Goodsell, A. J. Olson, *J. Comput. Chem.* **2009**, *30*, 2785–2791.

The binding of RVD-127 in the binding site of the clathrin is shown in Figure S2b. The protein is shown as opaque surface (olive-green), the docked RDV-127 as stick model colored by element, and the transparent red surface depicts the original Pitstop-2 from experimentally determined crystal structure.


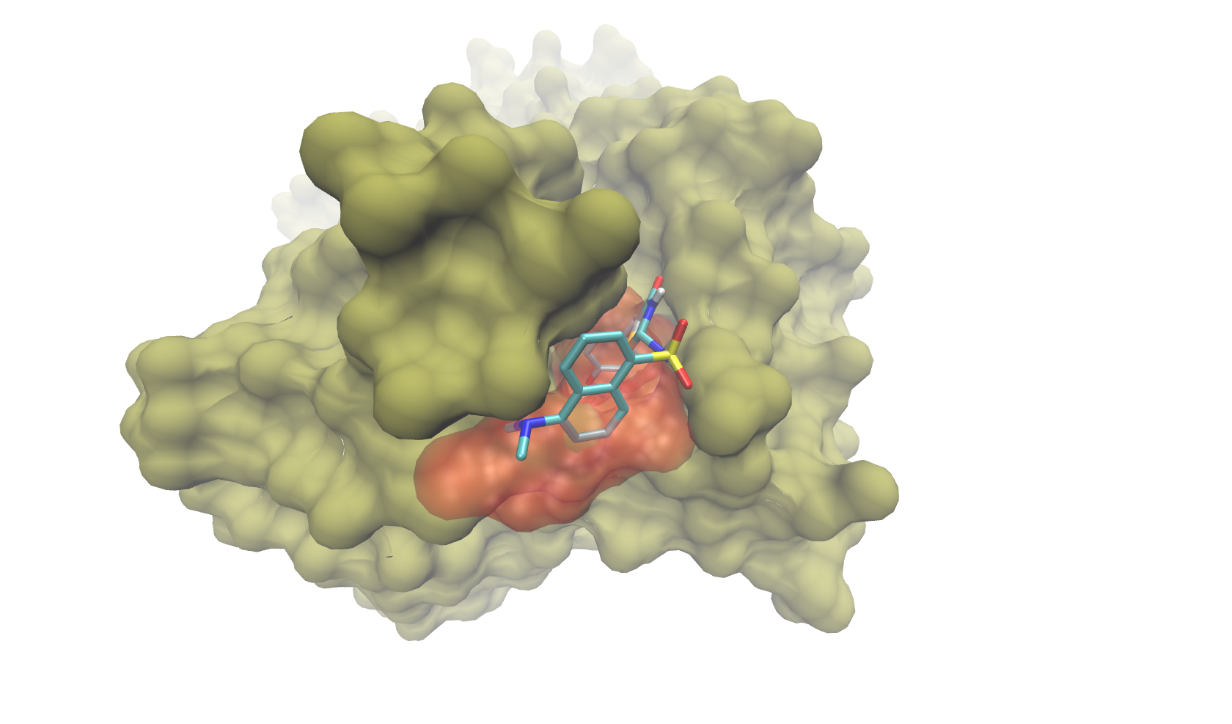


**Figure S2b.** The binding of RVD-127 in the N-terminal domain of clathrin.

As seen from both Figures S2a and S2b, the binding positions of RVD-127 and the unmodified Pitstop-2 are very similar. They both occupy the same binding pocket and the orientations of the naphthalene and the styrene moieties within the pocket are almost identical, with only a slight difference in the orientation of the pseudothiohydantoin ring. From these observations, we hypothesized that RVD-127 will show a similar bioactivity as the lead structure.

**Figure S3**


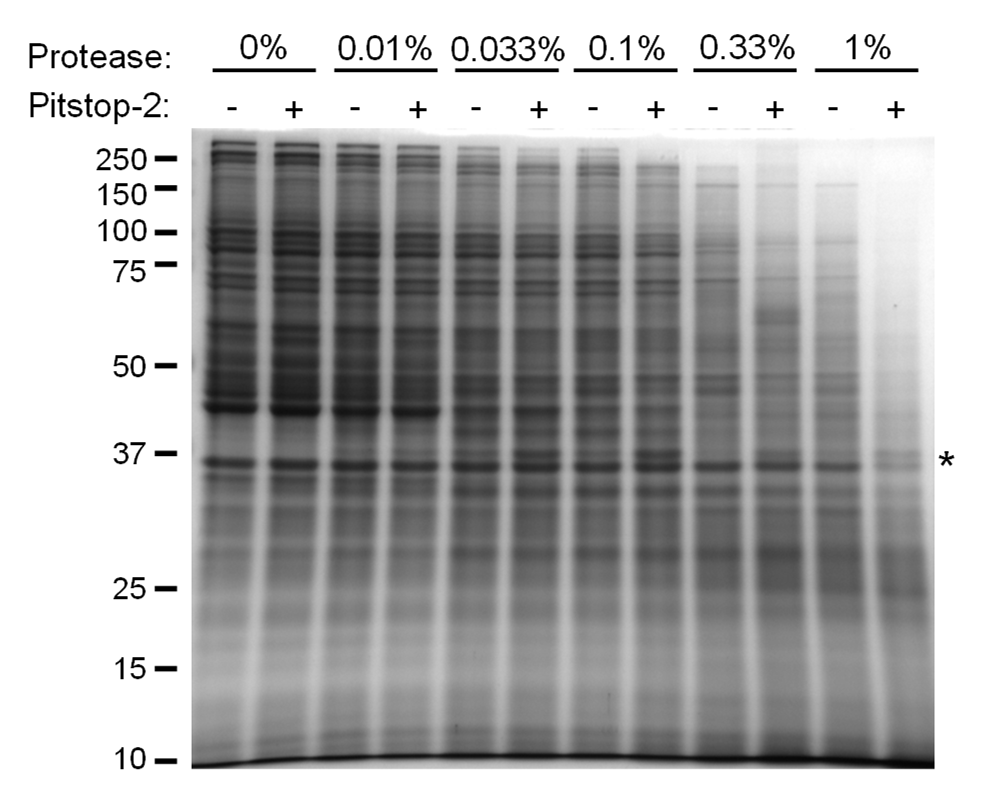


**Figure S3.** Drug affinity responsive target stability (DARTS) assay for the identification of the protein target of Pitstop-2. DARTS of EA.hy 926 cell extracts pre-incubated with Pitstop-2 or solvent (Dimethyl sulfoxide, DMSO) show a single prominent protein band composed mainly of cytoplasmic β-actin protected by the drug from proteolysis (marked by asterisk). N = 3.

**Figure S4**


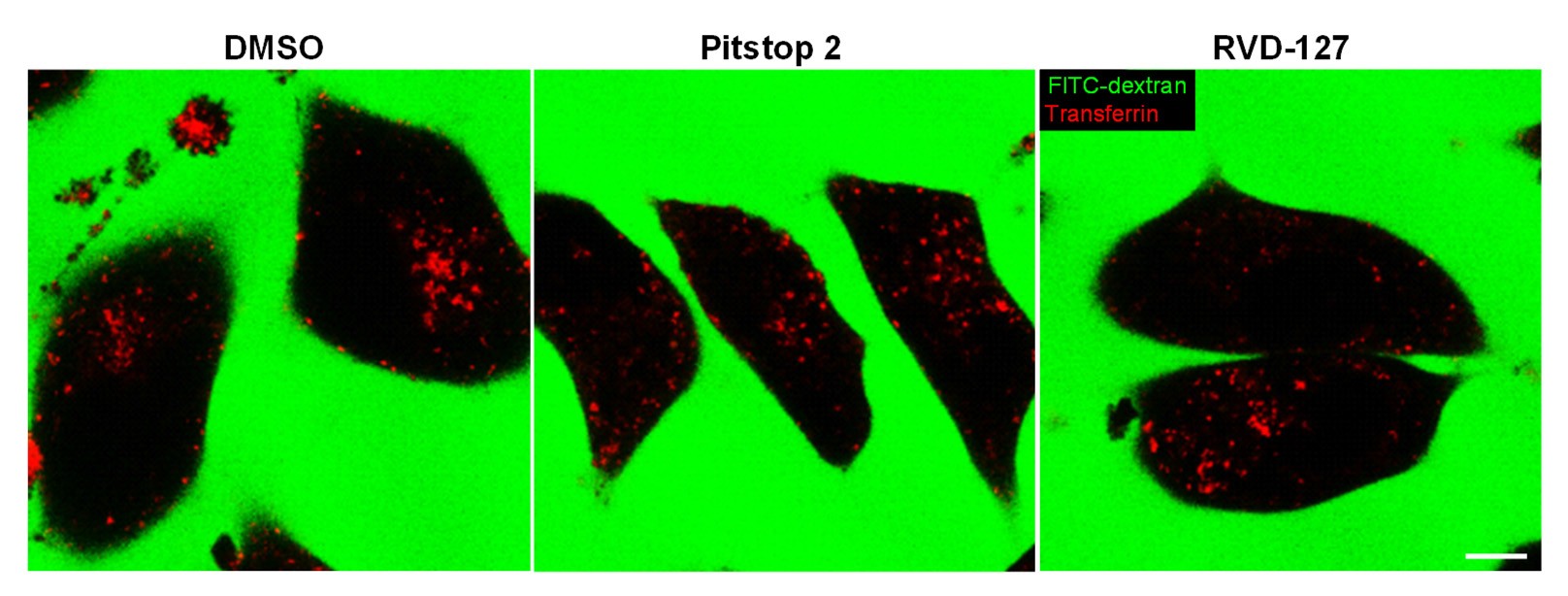


**Figure S4.** 7.5 μM Pitstop-2 and 50 μM RVD-127 are barely able to inhibit clathrin-mediated uptake of transferrin, if at all, in contrast to their highly inhibitory effects at concentrations of 100 µM and 30

µM, respectively (Figure 1). Scale bar = 10 μm. N = 3.

**Figure S5**


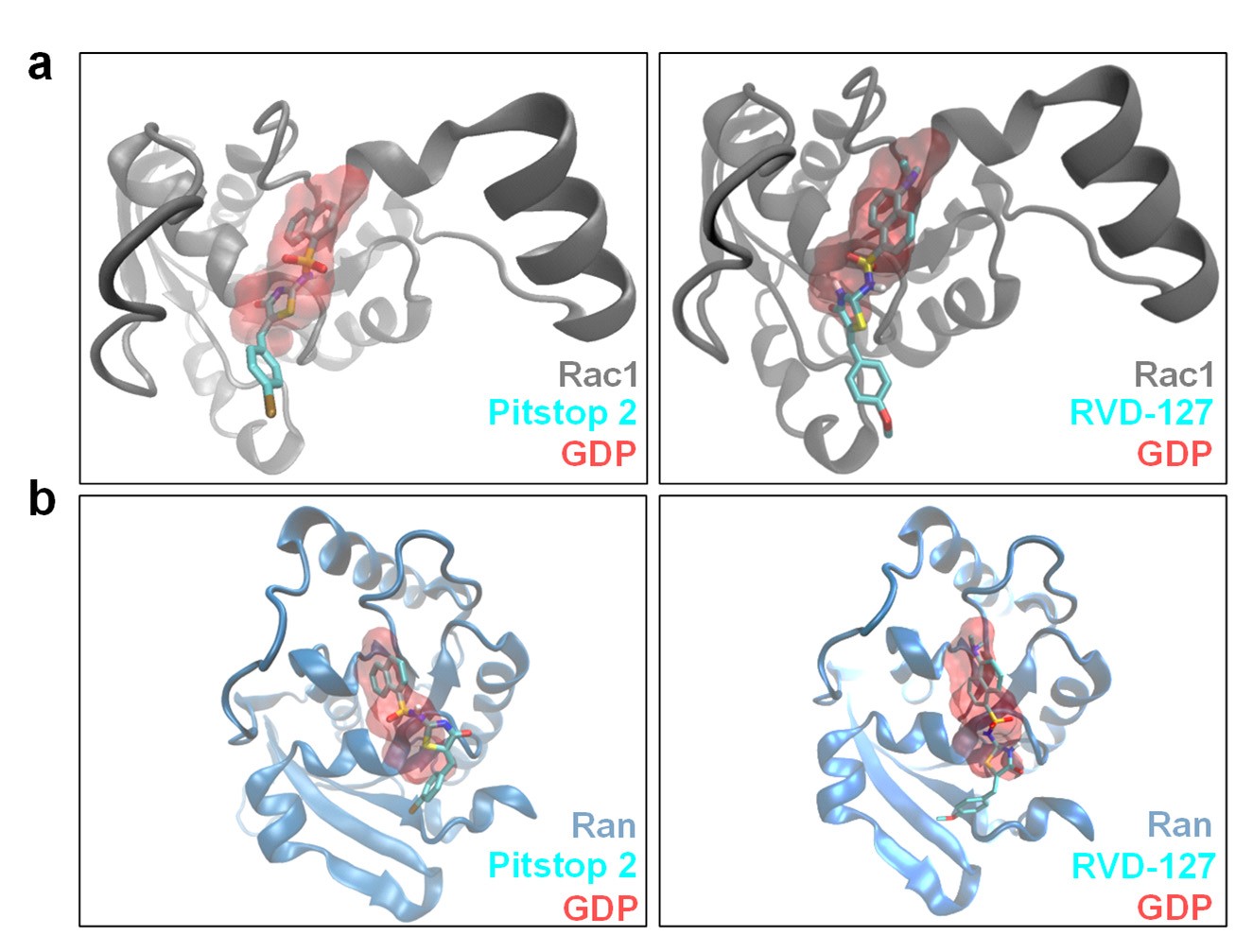


**Figure S5.** Molecular simulation of RVD-127 binding to Rac and Ran (GDP pocket)

# Supporting movie captions:

## Movie S1

AFM imaging of an endothelial cell dynamics prior to exposure to Pitstop-2. Framerate = 2 images per minute.

## Movie S2

AFM imaging of an endothelial cell dynamics upon exposure to Pitstop-2. Lamellipodial arrest and gradual dismantling of cortical actin network are readily apparent. Framerate = 2 images per minute.

## Movie S3

DIC imaging of an endothelial cell dynamics prior, during and after wash-out of DMSO (Dimethyl sulfoxide, solvent for Pitstop-2 and RVD-127). Framerate = 2 images per minute.

## Movie S4

DIC imaging of an endothelial cell dynamics prior, during and after wash-out of Pitstop-2. Framerate

= 2 images per minute.

## Movie S5

DIC imaging of an endothelial cell dynamics prior, during and after wash-out of RVD-127. Framerate

= 2 images per minute.
